# Knowledge, Attitudes, and Practices of Military Personnel Regarding Heat-Related Illness Risk Factors: Results of a Chinese Cross-Sectional Study

**DOI:** 10.3389/fpubh.2021.707264

**Published:** 2021-06-25

**Authors:** Xuren Wang, Demeng Xia, Xisha Long, Yixin Wang, Kaiwen Wu, Shuogui Xu, Li Gui

**Affiliations:** ^1^Emergency Nursing Department, School of Nursing, Naval Medical University, Shanghai, China; ^2^Nursing Department, The Second Naval Hospital of Southern Theater Command of PLA, Hainan, China; ^3^Department of Emergency, Changhai Hospital, Naval Military Medical University, Shanghai, China; ^4^Department of Orthopaedics, The Naval Hospital of Eastern Theater Command of PLA, Zhejiang, China; ^5^Southwest Jiaotong University College of Medicine, Southwest Jiaotong University Affiliated Chengdu Third People' s Hospital, Chengdu, China

**Keywords:** heat wave, heat-related illness, military personnel, China, knowledge, attitude, practice

## Abstract

**Background:** Military personnel are widely exposed to risk factors for heat-related illnesses. Knowledge, attitudes, and practices (KAP) are three of the most important means by which to prevent such illnesses, but there has been a lack of investigations into and correlation analyses of KAP. This study aimed to explore the heat-related KAP of military personnel in China.

**Methods:** We conducted a cross-sectional study (June 1-25, 2019). A total of 646 military personnel were recruited from two Chinese Navy troops in the tropical zone and one troop in the temperate zone. We collected data on demographic characteristics and KAP scores using questionnaires. Univariate analysis and Scheffe's method were used for data analyses.

**Results:** The mean KAP scores were 10.37 (range = 3–13, standard deviation = 1.63) for knowledge (K-score), 7.76 (range = 0–16, SD = 2.65) for attitudes (A-score), and 3.80 (range = 1–6, SD = 1.12) for practices (P-score). There were noticeable differences in mean K-score according to age, military rank, and educational level (*P* < 0.05). Participants from the tropical zone had higher A-scores (*P* < 0.05) and higher P-scores (*P* < 0.001) than those from the temperate zone. Additionally, participants with relevant experience also had higher A-scores (*P* < 0.05) than those without such experience.

**Conclusions:** Military personnel's awareness of preventive and first-aid measures against heat-related illnesses need to be strengthened. It will be very important to develop educational programmes and enrich systematic educational resources to raise this awareness.

## Highlights

- Since military personnel are widely exposed to risk factors for heat-related diseases, and this is the first time that the Chinese Navy has investigated KAP for heat-related diseases, research on this population may be of great significance.
- Military personnel scored highly on most common-sense questions about heat-related illnesses,but the accuracy rate of questions about exertional heat stroke were extremely low.
- Some misinformation related to media consumption with commercial purpose may be fatal at the critical moment for rescuing severe exertional heat stroke patients.
- The majority of respondents had good awareness of heat-related illnesses,and those from the humid tropical zone had higher mean A-scores than the temperate zone.
- Pearson's correlation coefficient indicated a weak correlation between the A- and P-scores.

## Background

The Intergovernmental Panel on Climate Change (IPCC) projects that the frequency, duration, and intensity of extreme weather may increase in the coming decades (1). A heat wave (HW) is a natural hazard characterized by an episode of hot weather. However, there is currently no universally accepted definition of HWs around the world in different fields. Especially in the military system, various definitions are employed (2). Thus, given this divergence, this study adopted the definition of three or more consecutive days with a maximum temperature over 35°C as published by the Chinese Meteorological Administration (3). The frequency of heat waves has increased in most parts of Asia (4), Europe (5, 6), and Australia (7, 8). Furthermore, heat waves can have significant effects on health and present a challenge for occupational-health protection. Heat-related illnesses include heat stroke, heat exhaustion, rhabdomyolysis, heat spasm, heat syncope, and heat rash. The inverse effects of heat-related illnesses on mortality have been widely reported. Mortality from heat stroke among the elderly exceeds 50% (9). Another study, conducted in 66 cities in China, showed that 5.0% of excess deaths may be associated with heat waves (10). The estimated number of heat-related deaths worldwide is expected to increase to 90,000 annually in 2030 and more than 255,000 in 2050 (11). Therefore, more attention should be paid to the insidious health effects of heat-related illnesses.

Risk factors associated with heat-related illnesses may be environmental or individual. Environmental risk factors, also known as exogenous factors, may include high temperatures, high humidity, and direct sun exposure. Individual risk factors, also known as endogenous factors, may include insufficient fluid intake, physical exertion, overall physical condition, medications, and pregnancy (12). Military personnel, especially those at low latitudes, where soldiers routinely experience high levels of physical exertion under high ambient temperatures and high humidity, are widely exposed to both exogenous and endogenous risk factors for heat-related illnesses. Military endeavors in heat wave conditions can alter the judgement and physical performance of military personnel, leading to significant impairment of individuals' ability to work, possibly even leading to death (13, 14). Therefore, reducing heat-related illnesses is a key factor in ensuring the combat effectiveness of the military during heat waves.

The purpose of knowledge, attitudes, and practices (KAP) surveys is to collect data on the knowledge, perceptions, and behaviors of specific populations in relation to a certain topic. The literature shows that knowledge of heat waves, attitudes toward risk factors, and adaptation practices are three of the most important factors in preventing heat-related illnesses (15). These findings could merely be local indicators that are representative of a particular field. KAP studies on heat-related illnesses have been performed among the general public for different occupations, and it is reported that several factors influence public KAP, such as age, educational level, economic level, nationality, and gender (16–18). However, only a few studies have focused on knowledge of heat-related illnesses among Chinese military personnel. There is a lack of investigations and correlation analyses of knowledge, attitudes, and practices. Therefore, in this study, we selected three Chinese naval troops with different risk factors that were working at low latitudes to explore the heat-related KAP of military personnel for the first time. Our aim was to provide data for future policy formulation and implementation in response to heat waves and associated side effects.

## Methods

### Study Area and Participants

**Figure 1:**
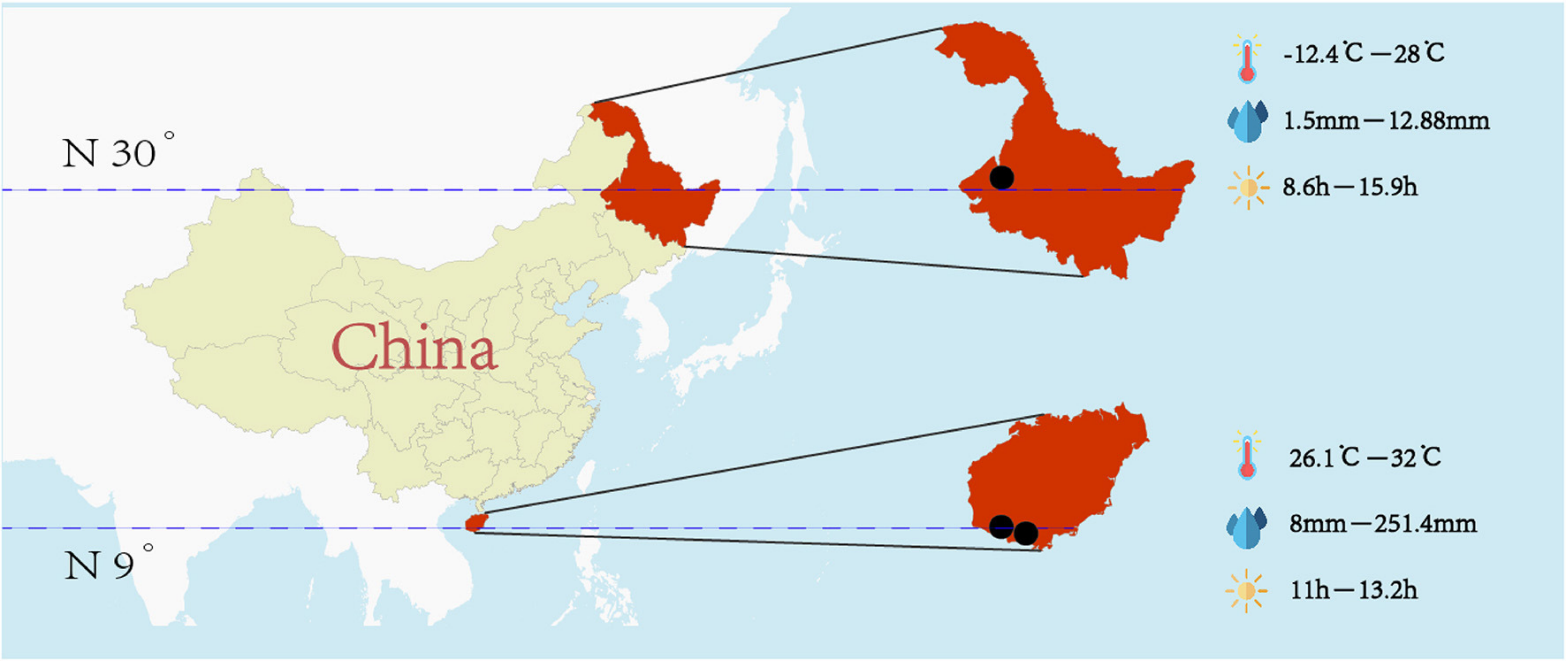
Geographical location and climatic characteristics of the three sample troops.

A total of three naval troops took part in the study. Two of these troops were stationed in the tropics (~9° north latitude), where they worked in a high-temperature and high-humidity environment all year round. The hottest month (in terms of average maximum temperature) was May (32°C). The month with the lowest average temperature was January (26.1°C); the wettest month (with the most rainfall) was September (251.4 mm), and the driest month (with the least rainfall) was January (8 mm). The month with the longest sunshine duration was June (average sunshine duration: 13.2 h). The month with the shortest sunshine duration was December (average sunshine duration: 11 h). The other sampled troop was stationed in a warm, temperate, continental monsoon climate zone (~30° north latitude). The hottest month (with the highest average temperature) was July (28°C). The month with the lowest average temperature was January (−12.4°C). The wettest month (with the most rainfall) was July (128.8 mm). The driest month (with the least rainfall) was January (1.5 mm). The month with the longest sunshine duration was June (average sunshine duration: 15.9 h). The month with the shortest sunshine duration was December (average sunshine duration: 8.6 h). The geographical location and climatic characteristics of the three troops mentioned above was shown in Figure 1.

We used convenience sampling to select military personnel from these three naval troops. The target population of this study was active-duty sailors without experience working in health care. A platoon is a military unit containing 30–50 sailors. We included a total of 15 platoons of troops in the tropical zone, including 560 sailors in total, in this study. In the temperate zone, we included three platoons with a total of 86 sailors.

### Data Collection

We developed a questionnaire based on a review of the literature on heat waves and heat-related illnesses (Supplementary Material 1). The questionnaire “Research Questionnaires on knowledge, attitude and practice toward heat-related illnesses during field training exercises” was initially drafted in English by Li Gui and Sarathchadra, and was translated from English to Chinese by Demeng Xia, Xuren Wang, and then was translated then back to English by Yixin Wang, Xisha Long to ensure the meaning of the content. The questionnaire consisted of four sections: (1) sociodemographic information, including age, years of military service, educational level, marital status, military rank, and heat-related illnesses experience; (2) the knowledge (K) section including 18 items on clinical symptoms, treatment, risk factors, prevention and control of heat-related illnesses (13 true–false items and five multiple-choice items); (3) the attitude (A) section including four items about attitude of sailors toward heat-related illnesses; and (4) the practice (P) section including six items related to practices and behavior of heat-related illnesses prevention.

In the K section, participants received one point for answering each true–false item or multiple-choice question correctly; incorrect answers received zero points, with high scores indicated better knowledge of heat-related illness risk factors. Items in the A section were scored on a four-point scale, a high score indicates a positive attitude. The scale used Cronbach's α to assess internal reliability. Cronbach's alpha coefficient is 0.73, indicating internal reliability. The P section consisted of yes-or-no questions, with each “Yes” response earning one point and each “No” response earning zero points. The score ranges for the K, A, and P sections were 0–18, 1–16, and 0–6, respectively.

This cross-sectional quantitative survey collected data face-to-face using the time of regular assembly from 1 June 2019, to 25 June 2019. Well-trained researchers interviewed participants using the structured questionnaires, and respondents were informed that all information and opinions provided would be anonymous and confidential. Various actions were taken to ensure questionnaire quality. First, a panel of experts was consulted at the development stage, and then a pilot study including only a few sailors was carried out for semantic analysis. Before the survey, all the researchers were systematically trained in the unified interview guide and questionnaire instructions. All questionnaires were completed and collected immediately to increase the response rate. Two independent researchers performed data collation and entry to minimize errors in data processing.

### Data Analysis

We used SPSS for Mac software version 25.0 (IBM Corp., Armonk, New York, US) for data analysis. Mean and standard deviation (SD) values were calculated for continuous variables; categorical variables are expressed as the percentage of subjects. We used univariate analysis of variance to test the associations of each demographic characteristic with K-, A-, and P-scores and the overall score. Scheffe's method was used in further paired comparisons if necessary. Finally, we used Pearson's correlation coefficient to clarify the correlations between K-, A-, and P-scores. *P* < 0.05 was considered statistically significant.

### Ethical Considerations

The study was approved by the Ethics Committee of the Institutional Review Board of the Naval Medical University, Shanghai, China (NMUMREC-2021-022). Written informed consent was obtained from all participants before the survey. All data obtained were anonymous.

## Results

### Participant Demographics

In the baseline survey, a total of 646 subjects were approached and invited to join this study. However, six of them did not complete the questionnaires, leaving 640 (99.1%) in the final analysis. Their sociodemographic characteristics are presented in Table 1. All participants were male, and their mean age was 25.1 years (range = 18–43 years, SD = 4.09). The greatest share of participants (43.9%) had been in military service for 1–5 years. Educational levels and military ranks varied. Of all participants, 86.8% were stationed in the tropical zone, and 29.1% had heat-related illness experience.

**Table 1:** Demographic characteristics (*n* = 640).

**Characteristic**	**Category**	***n***	**Proportion (%)**
Age (years)	≤20	59	9.2
	21–25	347	54.2
	26–30	154	24.1
	≥30	78	12.2
	Unanswered	2	0.3
Years of military service	≤1	48	7.5
	1–5	281	43.9
	6–10	159	24.8
	11–15	107	16.7
	≥16	44	6.9
	Unanswered	1	0.2
Education level	Bachelor level or above	139	21.7
	Junior middle school	214	33.4
	Senior middle school	253	39.5
	Elementary school	31	4.8
	Unanswered	3	0.5
Marital status	Unmarried	479	74.8
	Married	157	24.5
	Divorced	2	0.3
	Unanswered	2	0.3
Rank	PFC	90	14.1
	Corporal	206	32.2
	Sergeant or above	248	38.8
	Junior officer	76	11.9
	Field officer	17	2.7
	Unanswered	3	0.5
Climate zone	Tropical zone	556	86.8
	Temperate zone	84	13.2
Heat-related illness experience	Yes	186	29.1
	No	453	70.8
	Unanswered	1	0.2

### Response to Questions on Knowledge

Table 2 details the responses showing participants' knowledge about heat-related illnesses. More than half of the true–false questions received correct answers from >80% of respondents. Most participants (95.9%) were familiar with heat exhaustion management, including transferring victims to a cool environment; drinking fluids; and using cool water, ice packs, and fanning. However, over one-third of participants (34.7%) did not know that sweating could reduce body temperature, and 37.5% of participants thought that only physically weak persons were susceptible to heat-related illnesses during field training exercises. Moreover, 81.2% of participants deemed that heat exhaustion is characterized by a body temperature higher than 40°C, which showed that most participants did not have basic knowledge of heat stroke. The multiple-choice items received far fewer correct answers than the true–false items did. Alcohol was considered by 77.4% of participants to be the best means of decreasing health risks from heat waves, when actually it is a risk factor. The World Health Organization (WHO) recommends drinking water or using oral rehydration salts (ORS) (19), but 85.0% of participants said that they preferred to drink soda during field training exercises.

**Table 2:** Responses to knowledge items (*n* = 640).

	**Question**	**Category**	***n* (%)**
Yes or No responses	1. Could fainting and collapse be due to heat-related illnesses during field training exercises?	Yes^a^ No	84.1 15.9
	2. Is heat exhaustion managed by transferring the victim to a cool environment, drinking fluids, and applying cool water, ice packs and fanning?	Yes^a^ No	95.9 4.1
	3. Are fever, fatigue, and chest tightness common symptoms of heat stroke?	Yes^a^ No	80.1 19.1
	4. When heat stroke is suspected, should you first transfer the victim to a cool environment and then ask for an ambulance?	Yes^a^ No	93.2 6.8
	5. Can wearing thick clothes prevent heat stroke?	Yes No^a^	5.5 94.5
	6. Could the victim's muscle cramps be caused by heat-related illnesses during field training exercises?	Yes^a^ No	81.8 18.2
	7. Can cooling the body down prevent heat stroke?	Yes^a^ No	86.5 13.5
	8. Can staying in cold spots prevent heat stroke?	Yes^a^ No	93.3 6.7
	9. Is dehydration one of the symptoms of heat stroke?	Yes^a^ No	92.2 7.8
	10. Can sweating lower body temperature?	Yes^a^ No	65.3 34.7
	11. Are only physically weak persons susceptible to heat-related illnesses during field training exercises?	Yes No^a^	37.5 62.5
	12. Can heat-related illnesses cause a rapid loss of the victim's life during field training exercises?	Yes^a^ No	84.0 16.0
	13. Is heat exhaustion characterized by a body temperature higher than 40 degrees?	Yes No^a^	81.2 18.8
Multiple-choice responses	1. Please select the symptoms or signs of heat-related illnesses that you consider to be severe during a field training exercise	No sweating^a^ Sweating Fainting Fatigue	25.2 78.2 38.8 20.5
	2. Which drink would you prefer for a heat victim during field training exercises?	Ginger drink Soda drink Water and ORS^a^ Coffee	40.3 85.0 36.8 22.7
	3. Which of the following factors increases the risk of heat-related diseases	Aging Overweight Alcohol Sufficient fluid intake^a^	21.6 29.2 77.4 9.7
	4. How can a person prevent heat-related illnesses during field training exercises?	Alcoholic beverages Enough water^a^ Wearing thick and dark clothes Using sunscreen	93.4 14.4 40.5 38.9
	5. Which type of heat-related illnesses is the most serious?	Heat cramp Heat exhaustion Heat stroke ^a^ Heat syncope	60.3 70.5 37.5 73.4

a *The correct answer*.

### Responses to Questions on Attitudes and Practices

In the attitudes section, only 26.2% of participants said they were very concerned about the risks of heat-related illnesses. Additionally, 40.4% reported they were somewhat sensitive to heat-related illnesses, whereas <12.8% said they were “not at all” sensitive. In the practices section, most participants (79.7%) reported that when a high-temperature alert was released, their leaders generally arranged outdoor activities at relatively cooler times, and medics took intervention measures (74.4%). Additionally, 71.9% of participants were aware that it is necessary to implement good preventive measures against heat-related illnesses. However, only 64.8% of participants had received health education prior to field training, and nearly three-quarters of participants (74.4%) said that they drank water only when they were thirsty (Table 3).

**Table 3:** Responses to attitude and practice items (*n* = 640).

	**Question**	**Category**	***n* (%)**
Attitude	1. Do you intend to take preventive measures against heat cramps, heat exhaustion and heat stroke before and during field training exercises if a high-temperature warning is released?	Very much	45.1
		Much	42.4
		Sometimes	8.6
		Not at all	4.0
	2. How much do you worry about the risk of heat-related diseases in field training?	Very concerned	26.2
		Little concern	44.5
		Not at all	21.8
		I don't know	7.5
	3. Do you consider yourself sensitive to extreme heat?	Very much	34.7
		Somewhat	40.4
		Not at all	12.8
		I don't know	12.1
	4. Do you think the medics raise enough awareness for extreme heat?	Too much	30.3
		Just enough	40.4
		Too little	20.1
		I don't know	9.1
	1. Will your leaders generally arrange outdoor activities at a relative cooler time when a high-temperature warning is released?	Yes	79.7
		No	20.2
	2. Before you go out for field training exercises, does your medics tell you how to prevent and cope with heat-related illnesses?	Yes	64.8
		No	35.2
	3. When you go out for field training exercises, do you implement good heat-related illnesses preventive measures?	Yes	71.9
Practice		No	27.9
	4. During field training exercises, do you pay more attention to the signs and symptoms of heat cramps, heat exhaustion, and heat stroke?	Yes No	63.1 36.9
	5. Do you drink water only when thirsty during field training exercises?	Yes	74.4
		No	25.4
	6. When your troops go out for field training exercises, do medics prepare good heat-related illnesses intervention measures, such as medications, fluids and temperature-decreasing devices?	Yes	74.4
		No	25.4

### Mean Scores for Knowledge, Attitudes, and Practices

**Figure 2:**
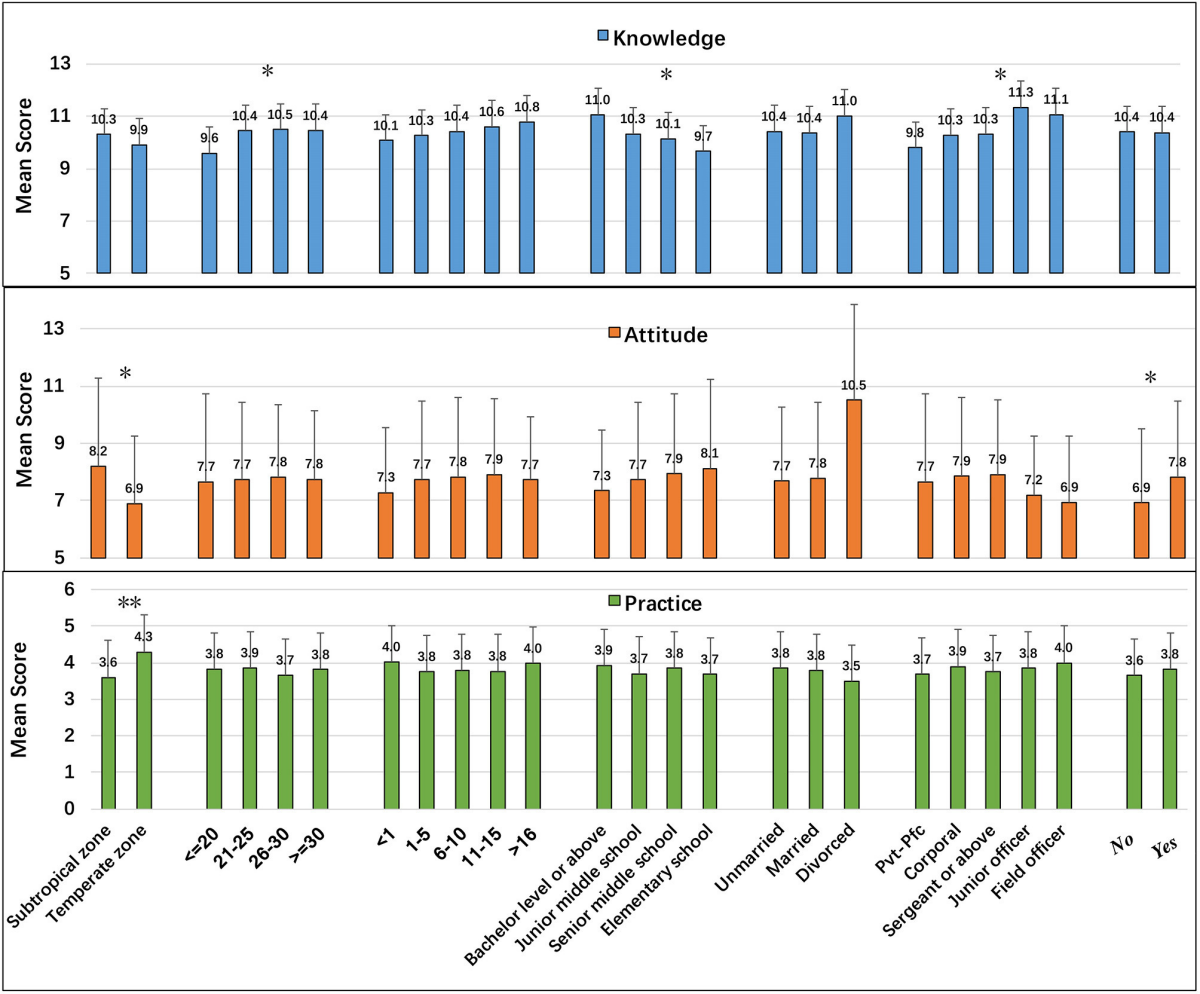
Mean KAP scores according to demographic characteristics. *P < 0.05, **P < 0.001.

Detailed mean KAP scores and mean overall scores according to demographic characteristics are shown in Figure 2. The mean K-score was 10.37 (range = 3–13, SD = 1.63). There were noticeable differences in mean K-score according to age, military rank, and educational level (*P* < 0.05). Paired comparisons using Scheffe's method indicated that the mean K-score was lower among participants <20 years old compared with the other age groups (*P* < 0.05) and higher among junior officers (*P* < 0.05) and participants who had at least a bachelor's degree (*P* < 0.05).

The mean A-score was 7.76 (range = 0–16, SD = 2.65). Participants from the tropical zone had higher A-scores than those from the temperate zone (8.2, SD = 3.08 vs. 6.9, SD = 2.34; *P* < 0.05). Participants with heat-related illness experience had higher A-scores than those who did not have such experience (7.8, SD = 2.65 vs. 6.9, SD = 2.58; *P* < 0.05).

The mean P-score was 3.80 (range = 1–6, SD = 1.12). Participants from the troops stationed in the tropical zone had higher P-scores (4.3, SD = 0.90 vs. 3.6, SD = 1.22; *P* < 0.001) than those from the temperate zone.

### Correlations Between Knowledge, Attitudes, and Practices

Correlation analyses suggested a significant positive correlation between A- and P-scores (*r* = 0.170, *P* < 0.001). No positive correlation was found between K- and A-scores or between K- and P-scores (Table 4).

**Table 4:** Correlations between knowledge, attitude, and practice scores.

**Variable**	**Knowledge score**	**Attitude score**	**Practice score**
Knowledge score	1		
Attitude score	0.004	1	
Practice score	0.020	0.170^**^	1

** *P < 0.001*.

## Discussion

Several studies have reported that heat waves have adverse effects on human health (19). People's awareness of the risks of, knowledge about, and protective practices against heat-related illnesses are crucial elements in reducing the harmful health effects of heat waves (20). However, to the best of our knowledge, this is the first study to survey KAP of heat-related illnesses in the Chinese Navy. Studies on this population could be greatly significant, as military personnel are widely exposed to risk factors for heat-related illnesses. Therefore, the findings of this study might provide essential references for the training and health education of military personnel.

In this survey, the majority of participants had high scores for most K-related questions and demonstrated good awareness of and protective practices against heat-related illnesses. However, some subgroups showed lower K-, A-, and P-scores based on demographic factors, environmental differences, and personal experiences.

Knowledge plays an important role in mitigating the adverse effects of heat waves (2). By analyzing participants' answers to K-questions in this study, we found that military personnel scored highly on most common-sense questions about heat-related illnesses (e.g., 95% of participants knew that being in a cool environment; drinking fluids; and applying cool water, ice packs and fanning were interventional measures against heat-related illnesses). However, the accuracy rate of questions about exertional heat stroke were extremely low. Only 37.5% of participants recognized the severity of exertional heat stroke, and only 25.2% of participants considered not sweating to be a danger sign. Exertional heat stroke is a medical emergency that is directly related to strenuous physical activity. Military personnel in high-temperature environments performing high-intensity exercise are vulnerable to exertional heat stroke (21). An epidemiological survey of military personnel showed a steady increase in the morbidity and mortality of exertional heat stroke over the past decade (22), but effective recognition and prompt treatment can greatly reduce this rate (23). Therefore, it is necessary to strengthen military personnel's awareness of how to prevent and administer first-aid in the event of exertional heat stroke.

Strikingly, 85% of participants chose soda as a drink for heat victims, and 93.4% of participants believed that alcoholic beverages were beneficial for preventing heat-related illnesses during field training. This misinformation that alcoholic beverages and soda contribute to the prevention and treatment of heat-related illnesses might be related to media consumption. Military personnel may access information via television, the Internet, and smart phones (24), but information from these media usually has a commercial purpose, which can mislead the audience. For example, advertisements often link ice-cold beer to hot summer weather and depict sportsmen in high-ambient temperatures delightedly drinking ice-cold soda. This erroneous information may be fatal at the critical moment for rescuing severe exertional heat stroke patients. According to a report in the New England Journal of Medicine, alcohol heightens the metabolic response to physical activity and is therefore a risk factor for exertional heat stroke. Thus, military administrators should strive to develop educational programmes in order to improve military personnel's knowledge about heat-related illnesses; moreover, governments should disseminate relevant knowledge on mass media.

By analyzing demographic characteristics, we found that participants who were younger than 20 years of age had lower K-scores than other participants (*P* < 0.05). This result was in contrast to the findings of Jing Li et al. (21). This might have been because participants in Jing Li's study had a large age range of 15–91 years, whereas the military personnel in our study were all young, with a mean age of 25.1 years. Additionally, K-scores were higher among junior officers (*P* < 0.05) with higher educational levels, which was similar to the results of previous studies (25). These findings suggest that we should provide health education on heat-related illnesses, especially to young military personnel. At the same time, since military officers have high educational levels and good mastery of relevant knowledge, administrators should take advantage of this, perhaps training these officers as instructors in health education projects.

The majority of respondents had good awareness of heat-related illnesses. Additionally, 87.5% of participants intended to take preventive measures at high ambient temperatures, and 75.1% of participants considered themselves sensitive to heat. Moreover, participants from the humid tropical zone had higher mean A-scores than those from the temperate zone. Conversely, when it came to the risks of heat-related illnesses, most participants (44.5%) reported little concern, possibly due to insufficient knowledge of these risks (26). Therefore, it is very important to enrich systematic educational resources with information about the risks of heat-related illnesses.

Pearson's correlation coefficient indicated a weak correlation between the A- and P-scores. This was consistent with the results of previous studies (27), whose authors reported that risk awareness is positively correlated with adaptation practices. An explanation for this correlation could lie in the health belief model, which asserts that health-related practices are determined by whether people recognize the seriousness of the problem and perceive themselves to be susceptible to particular illnesses (28). Therefore, good awareness of heat-related illnesses and the perception that there are benefits to taking action and fostering self-sufficiency against such illnesses promote preventive practices, which in turn reduce the adverse effects of heat waves. However, one subgroup in this study showed an interesting result: military personnel in the tropical zone had good awareness but low P-scores. This finding might be explained in part by three factors. First, people at low latitudes become better adapted to heat through behavioral and structural adjustment than people at high latitudes; this is called thermal acclimatization (29). Therefore, despite their positive attitudes toward heat-related illnesses, military personnel in the tropical zone performed limited protective behaviors. Second, the majority of participants were young men, who tend to be more willing than other people to take risks and to believe they can handle heat. Third, motivation and pressure from peers and instructors are likely to drive youths to perform beyond their physiological capability, which is also one of the major risk factors for exertional heat stroke (30). In summary, many factors might influence people's behavior, so further studies are needed to explore how to best promote and reinforce protective behaviors.

## Limitations

There were several limitations in our research. First, our present study investigated only KAP of heat-related illnesses in Chinese naval officers and sailors; thus, caution should be used when generalizing the results to other military forces. Second, this study adopted convenience sampling, which could limit the representativeness of the results. Third, the questions in the questionnaires relating to KAP of heat-related illnesses were limited rather than comprehensive and sufficiently detailed, meaning that we might not have explored the relevant knowledge mastery, behaviors, and attitudes in depth. Fourth, in the designed questionnaire, the answer options varied among questions. Specifically, there were three- and four-point Likert scales, which may have biased the research results. Finally but importantly, the sampling error was enlarged due to the difference in sample size between the two subgroups.

## Conclusion

Our research revealed that participants scored highly on most common-sense questions and demonstrated good awareness of and protective practices against heat-related illness. However, awareness of exertional heat stroke risks was inadequate. In addition, some differences, and personal experience. Thus, military personnel's awareness of preventive and first-aid measures against heat-related illnesses needs to be strengthened. To address these issues, it is very important to develop educational programmes and enrich systematic educational resources addressing heat-related illnesses.

## Data Availability Statement

The raw data supporting the conclusions of this article will be made available by the authors, without undue reservation.

## Ethics Statement

The studies involving human participants were reviewed and approved by the study was approved by the ethics committee of the institutional review board of the Naval Medical University. Written informed consent was obtained from all participants before the survey. All the data obtained was anonymous. The patients/participants provided their written informed consent to participate in this study.

## Author Contributions

LG and SX conceived and designed the study and administrative support. XW undertook data analysis, results interpretation, and manuscript preparation. DX and XL organized the field works and collected the data. YW and KW was responsible for critical revision of the manuscript. All authors contributed to the article and approved the submitted version.

## Conflict of Interest

The authors declare that the research was conducted in the absence of any commercial or financial relationships that could be construed as a potential conflict of interest.
