# Supplementary material for: Knowledge, Attitudes, and Practices of Military Personnel Regarding Heat-Related Illness Risk Factors: Results of a Chinese Cross-Sectional Study
Source: Front Public Health. 2021 Jun 25;9:707264. doi: 10.3389/fpubh.2021.707264 (PMC8267788; doi:10.3389/fpubh.2021.707264)
Supplement: Supplementary file 1 [file Table_1.DOCX]

**Research Questionnaires on knowledge, attitude and practice toward heat-related illnesses during field training exercises.**

**Demographical Data**

**Sex:** M ( ) F ( )

**Age Group:** 18-27 ( ) 28-37 ( ) 38-47 ( ) 48-57 ( ) 58-67 ( )

**Marital Status:** Unmarried ( ) Married ( ) Divorce ( ) Widowed ( ) Widower ( )

**Education Level:** High school graduate/ Vocational training ( ) Some University courses ( ) Bachelor’s degree ( ) Master’s degree ( )

**Branch of Military:** Army ( ) Coast Guard ( )

**Years of Military Service**: <1( ) 1-5( ) 6-10( ) 11-15( ) 16-20( ) >20( )

**Rank***:* Pvt-Pfc ( ) NCO ( ) Senior NCO ( ) Warrant Officer ( ) Junior Officer ( ) Company Grade Officer ( ) Field Grade Officer-General ( )

**Medical Educational Experience**: Yes ( ) No ( )

**Responses to questions on heat-related illnesses knowledge, attitude, and practice items.**

**Knowledge about heat-related illnesses. Yes or No responses.**

Please respond to the below listed questions by providing a check mark in the parenthesis:

1. Could fainting and collapse be due to heat-related illnesses during field training exercises? Yes ( ) No ( )

2. Is heat exhaustion managed by transferring the victim to a cool environment, drinking fluids, and applying cool water, ice packs and fanning? Yes ( ) No ( )

3. Are fever, fatigue, and chest tightness common symptoms of heat stroke? Yes ( ) No ( )

4. When heat stroke is suspected, should you first transfer the victim to a cool environment and then ask for an ambulance? Yes ( ) No ( )

5. Can wearing thick clothes prevent heat stroke? Yes ( ) No ( )

6. Could the victim’s muscle cramps be caused by heat-related illnesses during field training exercises? Yes ( ) No ( )

7. Can cooling the body down prevent heat stroke? Yes ( ) No ( )

8. Can staying in cold spots prevent heat stroke? Yes ( ) No ( )

9. Is dehydration one of the symptoms of heat stroke? Yes ( ) No ( )

10. Can sweating lower body temperature? Yes ( ) No ( )

11. Are only physically weak persons susceptible to heat-related illnesses during field training exercises? Yes ( ) No ( )

12. Can heat-related illnesses cause a rapid loss of the victim’s life during field training exercises? Yes ( ) No ( )

13. Is heat exhaustion characterized by a body temperature higher than 40 degrees? Yes ( ) No ( )

**Knowledge about heat-related illnesses. Multiple choice responses**

Please respond to the below listed questions by providing a check mark in the parenthesis assigned to the option:

1. Please select the symptoms or signs of heat-related illnesses that you consider to be severe during a field training exercise? No sweating ( ) Sweating ( ) Faint ( ) Fatigue ( )
2. Which drink would you prefer for a heat victim during field training exercises? Ginger drink ( ) Soda drink ( ) Water or ORS solution ( ) Coffee ( )
3. Which of the following factors increases the risk of heat-related diseases？Aging ( ) Overweight ( ) Alcohol ( ) Enough fluid intake ( )
4. How can a person prevent heat-related illnesses during field training exercises? Drink alcohol beverages ( ) Drink enough water ( ) Wear thick and dark clothes ( ) Use sunscreen ( )
5. Which type of heat-related illnesses is the most serious? Heat cramp ( ) heat exhaustion( ) heat stroke ( ) heat syncope ( )

**Attitude about heat-related illnesses.**

1. Do you intend to take preventive measures against heat cramps, heat exhaustion and heat stroke before and during field training exercises if a high-temperature warning is released? Very much ( ) Much ( ) Sometime ( ) Not at all ( )
2. How much do you worry about the risk of heat-related diseases in field training? Very concern ( ) Little concern ( ) Not at all concern ( ) I don’t know ( )
3. Do you consider yourself sensitive for extreme heat? Very well ( ) Somewhat ( ) Not at all ( ) I don’t know ( )
4. Do you think the medics raise enough awareness for extreme heat? Too much ( ) Just enough ( ) Too little ( ) I don’t know ( )

**Practice about heat-related illnesses. Yes or No responses.**  Please respond to the below listed questions by providing a check mark in the parenthesis:

1. Will your leaders generally arrange outdoor activities at a relative cooler time when a high-temperature warning is released? Yes ( ) No ( )
2. Before you go out in the field for training exercises, do your medics tell you how to prevent and cope with heat-related illnesses? Yes ( ) No ( )
3. When you go out in the field for training exercises, do you implement good heat-related illnesses preventive measures? Yes ( ) No ( )
4. During field training exercises, do you pay more attention to the signs and symptoms of heat cramps, heat exhaustion, and heat stroke? Yes ( ) No ( )
5. Do you drink water only when thirsty during field training exercises? Yes ( ) No ( )
6. When your troops go out for field training exercises, do medics prepare good heat-related illnesses intervention measures, such as medications, fluids and temperature-decreasing devices? Yes ( ) No ( )
